# Immunogenicity and functional characterization of *Leishmania*-derived hepatitis C virus envelope glycoprotein complex

**DOI:** 10.1038/srep30627

**Published:** 2016-08-02

**Authors:** Katarzyna Grzyb, Anna Czarnota, Agnieszka Brzozowska, Anna Cieślik, Łukasz Rąbalski, Jolanta Tyborowska, Krystyna Bieńkowska-Szewczyk

**Affiliations:** 1 Laboratory of Virus Molecular Biology, Intercollegiate Faculty of Biotechnology of the University of Gdańsk and Medical University of Gdańsk, Gdańsk, 80-307, Poland; 2 Laboratory of Recombinant Vaccines, Intercollegiate Faculty of Biotechnology of the University of Gdańsk and Medical University of Gdańsk, Gdańsk, 80-307, Poland

## Abstract

Hepatitis C virus (HCV) envelope glycoproteins E1 and E2 are the main inducers of a cross-neutralizing antibody response which plays an important role in the early phase of viral infection. Correctly folded and immunologically active E1E2 complex can be expressed in mammalian cells, though the production process might still prove restrictive, even if the immunological response of a vaccine candidate is positive. Here, we report a characterization and immunogenicity study of a full-length (fE1E2) and soluble version of the E1E2 complex (tE1E2) from genotype 1a, successfully expressed in the cells of *Leishmania tarentolae*. In a functional study, we confirmed the binding of both *Leishmania-*derived E1E2 complexes to the CD-81 receptor and the presence of the major epitopes participating in a neutralizing antibody response. Both complexes were proved to be highly immunogenic in mice and elicited neutralizing antibody response. Moreover, cross-reactivity of the mouse sera was detected for all tested HCV genotypes with the highest signal intensity observed for genotypes 1a, 1b, 5 and 6. Since the development of a prophylactic vaccine against HCV is still needed to control the global infection, our *Leishmania*-derived E1E2 glycoproteins could be considered a potential cost-effective vaccine candidate.

Hepatitis C virus (HCV) poses a significant health problem which affects an estimated 3% of the world’s population and causes approximately 500,000 deaths per year, coming as the result of HCV-associated liver diseases such as liver cirrhosis and hepatocellular carcinoma^1^. Despite progress in the development of highly effective direct-acting antivirals (DAAs), a prophylactic vaccine against HCV remains unavailable^2^,^3^. The development of a prophylactic cost-effective vaccine has proved challenging mainly because of high genetic diversity of HCV^4^. There are seven major genotypes and 67 subtypes with the differences going as high as 30–40% at the nucleotide sequence level^5^,^6^. The E1 and E2 envelope glycoproteins forming a heterodimeric complex exposed on the surface of the viral particle are genetically the most diverse HCV proteins. Their diversity stems mainly from constant humoral immune pressure directed against the E1E2 heterodimer^7^. Numerous reports indicate that humoral response plays an important role in the early phase of infection, and patients with high titers of the cross-neutralizing antibodies combined with a strong cytotoxic T cell response are able to combat and control the infection^8^,^9^,^10^. Importantly, the E1E2 complex is a potentially attractive vaccine antigen capable of eliciting a neutralizing antibody (nAbs) response. Recombinant forms of the envelope glycoproteins were the earliest prophylactic vaccine candidates against HCV. The most promising was the recombinant E1E2 complex expressed in the Chinese hamster ovary (CHO) cell line. In animal studies, immunized chimpanzees proved protected against chronic infection in an experimental challenge with homologous and heterologous HCV strains^11^. The vaccine reached phase I and was evaluated to be safe and generally well tolerated, inducing strong antibody and lymphoproliferative responses^12^. Further investigations support the use of recombinant E1E2 derived from genotype 1a as the vaccine antigen to induce broad cross-genotype neutralization^13^,^14^,^15^. Unfortunately, mammalian cell culture expression and glycoprotein purification turned out far too costly for commercial use. The whole purification procedure and technology of the vaccine candidate preparation must be acceptable in terms of the costs and scalable to the levels required for clinical trials and product marketing. That is why we still need alternative strategies for the recombinant E1E2 glycoprotein production.

Mammalian, insect and yeast expression systems offer the main eukaryotic platforms for the production of recombinant viral antigens today. Here, we investigated the possibility of expressing two forms of the E1E2 complexes, full-length (fE1E2) and truncated (tE1E2), in an unconventional *Leishmania tarentolae* expression system. The system was created in 2002 and is based on eukaryotic, mammalian-like protein folding and post-translational modification machinery. Moreover, *L. tarentolae* culture can be easily scaled up; hence the recombinant protein production yield can reach as much as several milligrams per liter of culture^16^,^17^. This report describes purification, functional characterization and immunogenicity of the *Leishmania*-derived HCV E1E2 complex.

## Results

There is multiple evidence indicating that most neutralizing epitopes are located primarily within the ectodomains of the E1 and E2 HCV glycoproteins^18^. In the past, several groups obtained the truncated forms of the E1E2 complex using insect, yeast, and mammalian expression systems^19^,^20^,^21^. In the present study, a similar soluble (tE1E2) construct devoid the transmembrane domains of E1 and E2 was designed, though an unconventional *L. tarentolae* eukaryotic expression system was applied for its expression. Full-length (fE1E2) and truncated (tE1E2) gene sequences of the HCV E1E2 complex were cloned into the *pLEXSY_I-blecherry3* vector and expressed in high-density protozoan cell cultures, using a tetracycline inducible expression system^22^. The production was performed in 1L shake flasks and lasted 72 h after tetracycline induction. Additionally, fE1E2 was expressed with its original signal peptide in contrast to tE1E2, where the original signal peptide was replaced with the *L. mexicana* signal peptide. As previously confirmed, application of the *L. mexicana* signal sequence facilitates secretion of the protein of interest into the culture medium^23^.

**Figure 1:**
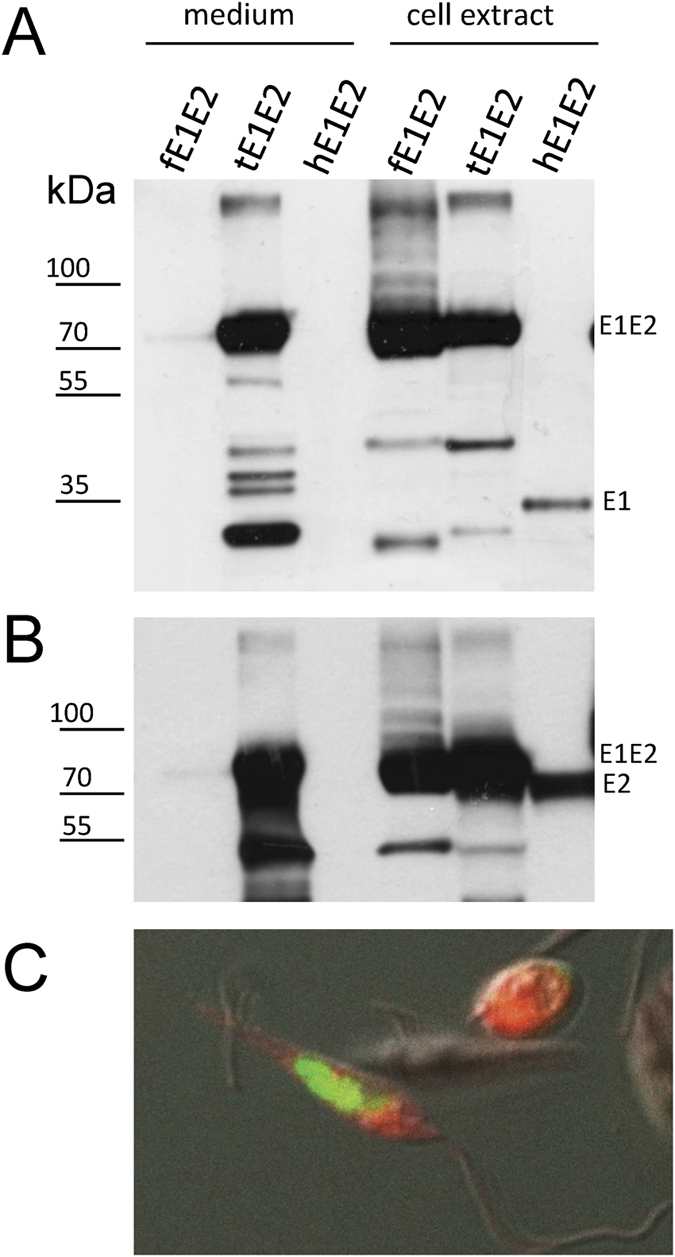
Analysis of the expression of the fE1E2 and tE1E2 complex by *L. tarentolae*. Western blot analysis of the E1E2 complexes detected in the medium and cell extracts using (**A**) anti-E1 Abs, and (**B**) anti-E2 Abs under reducing conditions. The E1E2 complex expressed in HEK 293 cells was used as the control (hE1E2). (**C**) *L. tarentolae* cell expressing the tE1E2 complex. Immunofluorescence with anti-E1 Ab (green); the red color corresponds to the Cherry fluorescence.

Protein expression was analyzed by immunofluorescence and western blotting of the culture medium and cell lysates using protein-specific anti-E1 and anti-E2 antibodies (Fig. 1A–C). The confocal microscopy confirmed that the E1E2 complex is predominantly located in the cytosol of the *L. tarentolae* cells, probably in the endoplasmic reticulum (ER) (Fig. 1C). Not surprisingly, only tE1E2 was efficiently secreted into the culture medium, although following detergent treatment a substantial amount of the protein was retained in the cell extract (Fig. 1A,B). In mammalian cells, full-length E1E2 is cleaved by a specific cellular protease into two separate proteins which assemble as non-covalent heterodimers retained mainly in the endoplasmic reticulum^24^. Strikingly, the fE1E2 complex expressed in the *L. tarentolae* is not properly cleaved unlike the E1E2 complex expressed in mammalian cells. In the western blotting analysis, anti-E1 and anti-E2 antibodies recognize the same band at the level of 80 kDa, which suggests that the cleavage between E1 and E2 does not occur (Fig. 1A,B).

**Figure 2:**
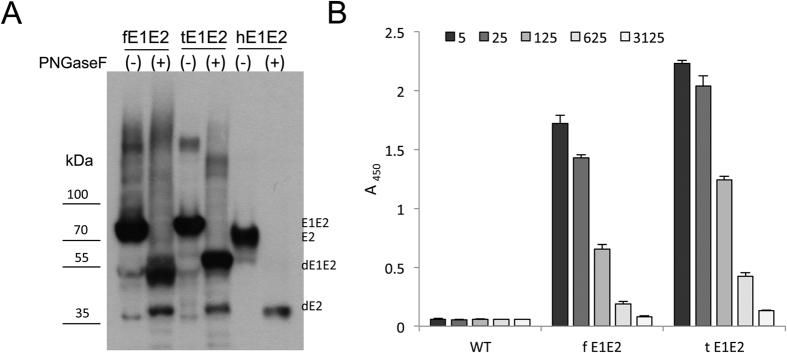
An N-glycosylation analysis of the fE1E2 and tE1E2 complex expressed in *L. tarentolae*. (**A**) The recombinant complexes were treated with endoglycosidase PNGaseF. After overnight incubation at 37 °C in native conditions, western blot under reducing conditions with anti-E2 Ab was performed. The E1E2 complex expressed in HEK 293 cells was used as the control (hE1E2). (**B**) Dose-dependent binding of the E1E2 complexes to GNA lectin. Decreasing concentrations of cell lysates (5-fold dilutions - from 1:5 to 1:3125) were captured in ELISA plates coated with GNA lectin. The bound antigens were visualized with anti-E2 Abs, anti-mouse IgG HRP conjugate, and the TMB substrate. The error bars represent the standard deviations of 2 replicate values.

The molecular weight of the *Leishmania*-derived E2 and E1 alone was assessed to be approximately 55 kDa and 28kDa, respectively. It is worth noting that the molecular weight of both *Leishmania*-derived E1E2 complexes was lower (approximately 80 kDa) compared to the E1E2 complex expressed in the HEK 293 cells (approximately 100 kDa). The explanation of the discrepancy may lie in the different N-glycosylation pattern. *Leishmania* is characterized by the absence of the higher-branched N-glycans, which may be the cause of the decrease in the molecular weight of the glycoproteins expressed in the *L. tarentolae* system versus the mammalian cells^16^. Despite the differences in the molecular weights, N-glycosylation of both complexes was confirmed by reaction with endoglycosidase PNGase F, where a decrease in the protein molecular weight (~25 kDa) after endoglycosidase digestion was observed (Fig. 2A). Furthermore, the binding to the *Galanthus nivalis* lectin was examined in GNA ELISA. A positive signal was detected at the lysate dilution of 1:625, which suggests that both complexes bound well to the lectin (Fig. 2B).

**Figure 3:**
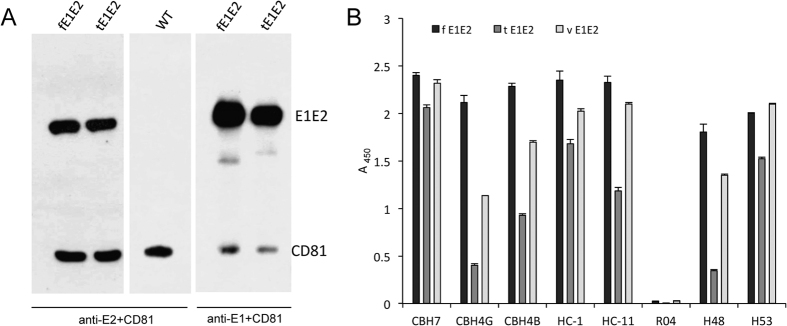
A functional analysis of the fE1E2 and tE1E2 complex expressed in *L. tarentolae*. (**A**) The GST-CD81-LEL pull-down assay. *L. tarentolae* cell wild-type lysate (WT) and lysates containing the recombinant E1E2 complexes were placed on glutathione–agarose beads preadsorbed with CD81-LEL fused to GST. After 16 h of incubation, the beads were washed and suspended in the SDS-PAGE sample buffer. Western blotting was performed with anti-E2, anti-E1, and anti-CD81 antibodies diluted 1:1000. (**B**) Analysis of the conformational epitopes of the fE1E2 and tE1E2 complex expressed in *L. tarentolae*. GNA-ELISA was performed with MAbs in the concentration of 5 μg/ml. R04 - isotype of the control MAb to a cytomegalovirus-specific protein. Huh 7.5 cell lysate containing gt1a/2a HCV chimera proteins was used as the positive control (vE1E2). The background from wild-type cell lysates was subtracted from obtained results. The error bars represent the standard deviations of 2 replicate values.

The CD81 receptor able to bind the HCV E2 glycoprotein is one of the important factors enabling HCV entry into the host cell. In this study, the binding of both fE1E2 and tE1E2 to the CD81 receptor was confirmed by a pull-down assay using recombinant CD81-LEL (Fig. 3A). Furthermore, we performed an analysis of conformational epitopes by detection with a panel of previously described human monoclonal antibodies specific to conformational epitopes on the E2 glycoprotein^25^,^26^. We performed characterization of the epitopes selecting HMAbs to domains A, B, and C of the E2 glycoprotein. Interestingly, the recombinant full-length complex (fE1E2) was recognized by all tested MAbs at the concentration of 5 μg/mL suggesting the presence of the crucial conformational epitopes. Surprisingly, the tE1E2 complex was mostly recognized by the CBH-7, HC-1, HC-11 antibodies found to be extensively HCV-neutralizing (Fig. 3B). The interaction of tE1E2 with CBH-4G and CBH-4B was weaker than the respective interaction of fE1E2, though the antibodies themselves showed minimum or no virus neutralization activity^25^. Finally, a positive ELISA signal was recorded for the response of both complexes to the conformational-dependent mAb H53 directed against the E2 ectodomain^26^,^27^ (Fig. 3B). In summary, in spite of TMD deletion and no correct E1E2 processing, both recombinant *Leishmania*-derived complexes show the presence of the essential conformational and broadly neutralizing epitopes.

**Figure 4:**
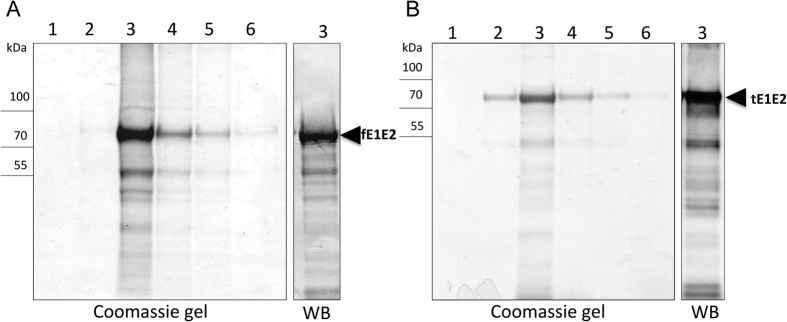
Purification of the fE1E2 and tE1E2 complex recombinantly expressed in *L. tarentolae.* (**A**) Purification of fE1E2 from the cell lysate on a Strep-Tactin column. (**B**) Purification of tE1E2 from the culture media on a Nickel column. The cells and culture media were collected 72 h after tetracycline induction. The recombinant protein induction process was performed in agitated cultures. The SDS-PAGE gels were stained with Coomassie R-250. Numbers 1–6 correspond to the elution fractions. The western blots (WB) were performed using anti-E2 Abs.

For the purpose of studying immunogenicity in mice both recombinant E1E2 complexes were purified in a one-step purification process as described in detail in the Methods section. The tE1E2 complex was purified directly from the cell culture medium using the Ni-NTA column. For purification of fE1E2, the Strep-Tactin column was used. Six elution fractions containing the fE1E2 and tE1E2 proteins were analyzed by SDS-PAGE and western blotting with anti-E2 antibodies. The purity of the main elution fraction was estimated at 80–90% by the SDS-PAGE method and western blotting analysis (Fig. 4A,B). The yield of the purification process was approximately 1 mg per liter of the cell culture. In spite of the presence of the secretion signal peptide from *L.mexicana*, almost half of the tE1E2 protein was retained in the cells. The purification process of the tE1E2 complex from cell lysates based on His-tag was not efficient (results not shown). Hence, only the tE1E2 complex purified from the cell culture medium was used for the immunization study.

**Figure 5:**
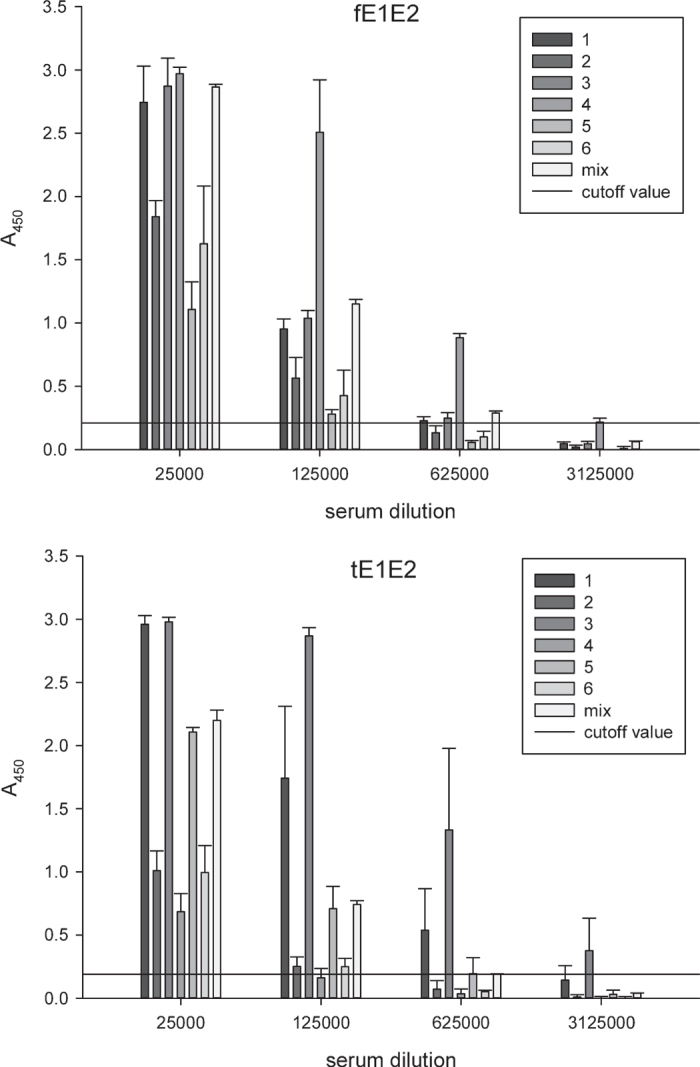
Antibody response to the fE1E2 and tE1E2 antigens used in mouse immunization. The background from the negative control serum in each dilution was subtracted from the obtained results. The titers were defined as the maximum reciprocal serum dilution able to recognize the antigen above the cut off value (triplication of the background A_450_ value). The data represent as the mean values from 2 independent experiments performed in duplicate.

To demonstrate immunogenicity of the recombinant E1E2 complexes, BALB/c mice were immunized three times subcutaneously on days 0, 21, and 42, in the presence of squalene-based oil-in-water nanoemulsion adjuvant. Primary immunization was performed using 10 μg of the recombinant proteins, while in the boosts the amount of protein was reduced to 5 μg. Blood samples were collected 2 weeks after the last vaccination. The serum antibody titer was determined by a set of the ELISA tests on the antigens used for mouse immunization and was defined as the highest serum dilution resulting in the absorbance value (A_450_) 3 times the background value. The terminal average serum titration showed that immunization with fE1E2 and tE1E2 resulted in high antibody titers reaching 6.25 × 10^5^ (Fig. 5).

**Figure 6:**
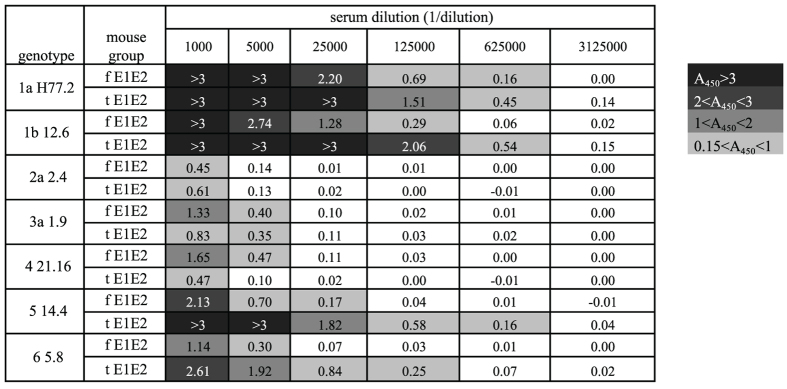
Cross-reactivity of the immune sera to the native E1E2 complexes from different HCV genotypes expressed in mammalian cells (HEK 293). The data represent the mean values from 2 independent experiments performed in duplicate. The background from the control serum in each dilution was subtracted from the obtained results. Dark gray indicates the highest signal intensity and the strongest binding, while white sections visualize the lowest intensity and no appreciable binding.

**Figure 7:**
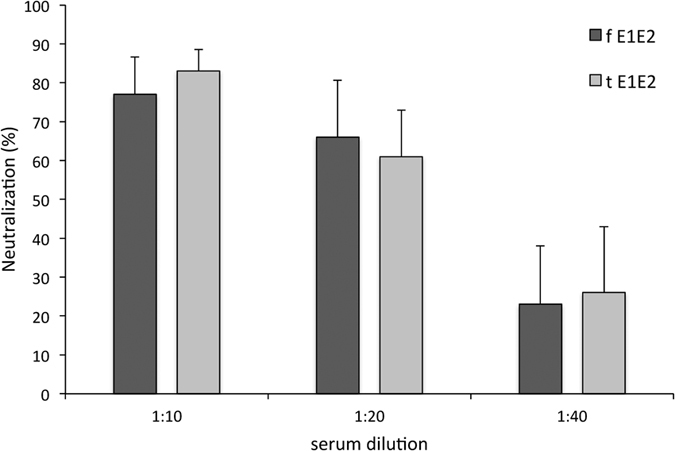
Neutralizing properties of mouse sera after immunization with fE1E2 and tE1E2. Pooled sera from 6 mice per group at dilution 1:10, 1:20 and 1:40 were incubated 2 h at 37 °C with HCVcc derived from genotype 2a. Infection levels were analyzed after 72 h by staining with anti-core antibody. Infected cells were counted under a microscope. The percent of neutralization was determined by comparing infectivity in the presence of post-immune serum with the infectivity in the presence of pre-immune serum at the same dilution. Bars represent the mean number of core-positive cell clusters counted from at least 6 independent microscope fields.

Since HCV displays a high degree of genetic diversity and variability we decided to explore cross-reactivity of the mouse sera to the native mammalian wild-type E1E2 from HCV genotypes other than those used for immunization. The immune sera were tested in 5-fold dilutions from 1:1000 to 1:3125000 (Fig. 6). As expected, the immune responses were the highest against genotype 1 and included not only the H77 homologous strain, but also the UKN 12.6 strain from genotype 1b. A relatively high signal was also obtained for genotypes 5 and 6. The weakest reactivity was observed for genotypes 2a, 3a. Of note, the sera of the tE1E2 group had a stronger affinity to genotypes 1a, 1b, 5, and 6. On the other hand, the reactivity of the sera of the fE1E2 group was slightly higher with genotypes 3a and 4. In addition, we evaluated the neutralizing potency of mouse sera in an infection assay using HCV cell culture system (HCVcc). As shown in Fig. 7, the number of HCV-positive cells was significantly reduced when the virus was incubated with both, fE1E2 and tE1E2 mouse sera compared to pre-immune serum. The results indicate that the immunized serum can neutralize genotype 2a virus in dose-dependent manner.

In general, the results indicate that both recombinant E1E2 complexes expressed in the *L. tarentolae* expression system are highly immunogenic. Moreover, the immunization procedures were effective in eliciting a specific and cross-reactive anti-E1E2 antibody response in mice.

## Discussion

A vaccine platform aimed at inducing an antibody response requires exposing the crucial neutralizing epitopes of the potential antigens. It is known that the E1E2 complex is an attractive candidate for a prophylactic vaccine against HCV. However, the E1 and E2 glycoproteins are heavily glycosylated and stabilized by numerous disulfide bridges with C-terminal transmembrane domains (TMD) anchored in the lipid envelope^27^,^28^,^29^. Moreover, their maturation process is slow and complicated, involving various chaperones of the host-infected cell^30^. These features make their recombinant production for vaccination purposes extremely difficult. High viral diversity poses another challenge when developing vaccine eliciting antibodies capable of neutralizing various HCV genotypes. However, it has previously been shown that vaccine comprising a recombinant E1E2 complex derived from a single genotype 1a strain is able to elicit cross-neutralizing antibodies in chimpanzees, healthy human volunteers and goats^13^,^14^,^15^. In this study, we provide the first evidence that expression of a functional and immunogenic E1E2 complex in protozoan *L. tarentolae* is possible. The most important advantage of the *L. tarentolae* system comes down to the ease of scaling up the cell culture and the possibility to perform further growing in biofermenters, the same as those used for prokaryotic cells. This makes the system attractive for industrial-scale protein production^17^. Only a few viral antigens have been previously expressed successfully in the *L. tarentolae* system– the truncated hepatitis E virus capsid protein^31^, the HIV-gag protein^32^, hemagglutinin (HA)^33^, and a soluble version of the Hendra virus attachment protein (HeV G)^34^. In the last three approaches, high immunogenicity of the *Leishmania*-derived products was demonstrated on animal models.

Here, using the *L. tarentolae* inducible expression system fE1E2 was expressed with its native signal sequence in contrast to the truncated form of the E1E2 complex (tE1E2) for which the signal sequence from the *L. mexicana* was used. Pion and his co-workers noticed that application of the *L. mexicana* signal sequence impaired the production of the HA proteins significantly^33^. In another study, no secretion of the soluble HeV G protein into the culture cell supernatant was detected^34^. Contrary to the above, in our study a soluble version of the E1E2 complex (tE1E2) fused with the *L. mexicana* signal peptide was efficiently produced and successfully secreted into the culture medium. Importantly, we did not optimize the codon use for the *L. tarentolae*. There are several reports providing evidence that optimized gene sequences, for example HA, allow attaining better protein expression^33^. To our knowledge, ours is the first report presenting application of a protozoan expression system for HCV E1E2 complex production, and further optimization studies are needed to improve the efficiency of the expression.

According to the earlier studies, the N-glycosylation pattern is highly variable in different *Leishmania* and *Trypanosoma* species. Most N-linked glycans are of the high mannose and pauci-mannose type, though many trypanosomatids have the enzymatic machinery which enables the formation of more complex N-glycan structures^35^. The N-glycosylation profile of *L. tarentolae* was investigated using recombinant human erythropoietin (EPO). The N-glycan structural analysis showed that the N-glycosylation pathway of the *L. tarentolae* was able to produce higher-eukaryote-like biantennary N-glycans, where only sialylation is missing^16^. The ectodomains of the HCV E1 and E2 glycoproteins are heavily glycosylated. E2 contains up to 11 N-linked glycosylation sites, most of them highly conserved across different genotypes. These glycans are essential for the proper folding and function of the E1E2, and play an important role in the viral entry^36^. Interestingly, glycosylation state is also known to be important for immunogenicity, the fact was proved for the HA protein^37^. In this study, both recombinant fE1E2 and tE1E2 complexes were glycosylated as shown in the PNGase F profile. However, the molecular weight of the *Leishmania*-derived E1E2 complex was below that of the E1E2 complex expressed in mammalian cells. This could be caused by lack of N-acetylglucosaminyltranferase IV-activity in *Leishmania* and the resulting absence of higher branched N-glycans^16^. Another striking feature of the E1E2 complex expressed in *L. tarentolae* is the absence of the signal protease specific for E1 and E2 processing. In general, the E1 and E2 structural glycoproteins are released from the viral polyprotein by the host cellular signal peptidases. The cleavage signal peptide of E2 corresponds to the E1 C-terminus^38^. It has been reported that *L. tarentolae* is potentially able to process signal sequences and express correctly folded proteins stabilized by disulfide bridges. For example, human laminin – disulfide-linked heterotrimeric glycoprotein - was successfully obtained in the *L. tarentolae* expression system^39^. However, in the full-length E1E2 complex expressed in the *L. tarentolae* we did not observe any cleavage between the E1 and E2 proteins which would be as efficient as that of the E1E2 complex expressed in mammalian cells. This could be explained by the absence of the signal protease specific for the E1 and E2 processing, or by lower efficiency of the protease activity in *L. tarentolae* versus the mammalian cells.

Previous studies have demonstrated that numerous neutralizing antibodies recognize regions on the E2 containing residues critical for binding to the host CD81 receptor^40^,^41^. CD81 is one of the crucial entry factors for the HCV and therefore the CD81-binding regions are highly conserved and represent the main target for a broad range of the nAbs. Moreover, studies of the E2 crystal structure have confirmed that the broadly neutralizing face of the E2 comprises a CD81 binding loop (aa519–535) and residues of the aa421–453, which makes those domains an attractive target for vaccine design efforts^42^. The recent data suggest that the recombinant E1E2 immunogen, previously applied in a phase I clinical trial, is capable of eliciting broadly cross-neutralizing Abs which targets the E2-CD81 interaction sites^15^. Human monoclonal antibodies HC-1, HC-11, and CBH-7 used in this study represent neutralizing antibodies directed against the antigenic domains B and C within the E2 glycoprotein which mediates in the binding to the CD81 receptor^9^,^43^,^44^. Proper recognition of the *Leishmania*-derived E1E2 complexes by these nAbs, as well as the binding to the CD81 receptor confirmed in a pull down assay, suggest that the exposition of the crucial cross-neutralizing epitopes is correct.

Lastly, several groups of researchers have already characterized the truncated forms of the E1E2 complex derived from different expression systems. The chimeric E1_341_E2_661_ complex obtained in insect cells was recognized by the conformation-dependent antibody H53 and human antibodies present in HCV-positive human sera^19^. In other approach, the E1_346_E2_699_ complex was expressed in the *Pichia pastoris* system. It was shown that the yeast-derived E1E2 truncated complex induced anti-E1E2 Abs in rabbits, the latter capable of neutralizing the HCVpp and JFH-1 derived virions^20^. Following a similar strategy, Ruwona and coworkers, designed a number of soluble E1E2 constructs. It was discovered that the E1_352_E2_717_ construct obtained by deletion of the transmembrane domains of both E1 and E2 was able to elicit a stronger than the full-length cross-reactive antibody response. It should be emphasized that mice were immunized with DNA constructs and despite the presence of antibodies against the conserved E2 antigenic regions, the serum antibody titer was low and displayed weak virus neutralization properties^21^. Interestingly, in our functional studies of the *Leishmania*-derived soluble tE1E2 complex, we confirmed the actual binding to the CD-81 receptor and the presence of the major epitopes participating in a broad neutralizing antibody response. It is well documented that full-length E1E2 is able to elicit a broad cross-neutralizing immune response across different HCV genotypes. In this report, we further confirm that soluble tE1E2 can also be considered a potential vaccine component eliciting a strong cross-reactive B-cell response, its breadth similar to that of the full-length E1E2.

Recent reports provide further support for the clinical development of the recombinant E1E2 complex as a promising vaccine antigen. It has been demonstrated that vaccine based on a single 1a genotype H77 strain expressed in mammalian cells is capable of eliciting cross-neutralizing antibodies which target multiple conserved regions of the E1E2^14^,^15^. Our data confirm actual cross-reactivity of the sera for all tested genotypes, though the highest signal intensity was observed for genotypes 1a, 1b, 5, and 6, and the lowest for genotypes 2a and 3. Since genotypes 2 and 3 are considered the most divergent from genotype 1, it was not surprising that the reactivity of the sera to these genotypes was the weakest. There is evidence indicating that recovery from HCV genotype 1 infection protects chimpanzees against infection caused by the other genotypes exhibiting up to 30% divergence at the amino acid level^45^. Recent studies show that in most cases immunization with E1E2 (H77) elicits antibodies of poor neutralizing potency against the 2a, 2b, and 3a genotypes^13^,^14^,^15^. Our cross-reactivity data are in line with those reports and confirm limited reactivity against genotypes 2a and 3a in contrast to genotypes 1a, 1b, 5, and 6. Nevertheless, despite the limited reactivity with E1E2 from genotype 2a in ELISA test, both groups of sera were able to neutralize HCV in cell culture. Certainly, the high titers of the cross-reactive and neutralizing sera obtained in this study are a promising contribution to further studies.

Ideally, an effective HCV vaccine should generate both humoral and cellular arms of the immune response. Recent reports suggest that a combination of envelope protein antigens with vectored vaccines should guarantee effective protection against HCV^46^,^47^. In conclusion, vaccination with *Leishmania*-derived E1E2 complexes combined with the squalene adjuvant resulted in cross-reactive and neutralizing antibody response in mice. High immunogenicity of the *L. tarentolae* products makes the system a promising vaccine platform for E1E2 industrial-scale expression.

## Methods

### Plasmid construction

To design the fE1E2 vector, the DNA sequence of full-length genotype 1a isolate H77 E1E2 (GenBank accession number, AF011751) was amplified by PCR and cloned into the *pLEXSY_I*-*blecherry3* expression vector (Jena Bioscience, Germany) using BglII and KpnI restriction sites. Next, the Twin-Strep tag DNA sequence was cloned into the same vector using KpnI and NotI restriction sites. The Twin-Strep tag amino acid sequence, WSHPQFEK-GGGSGGGSGGS-SAWSHPQFEK, added to the C-terminal sequence of E1E2 allowed efficient purification of full-length E1E2 from cell lysate supernatant (IBA, Germany). To generate the truncated form of the E1E2 complex (tE1E2), the nucleotide sequence of the ectodomains of E1 (aa192–352) and E2 (aa384–717) was synthetized without a linker (GeneArt) and cloned into the *pLEXSY_I*-*blecherry3* expression vector using XbaI and MspCI restriction sites for secretory expression. In *pLEXSY_I*-*blecherry3* expression vector blecherry gene allows selection with the antibiotic bleomycin and monitoring induction process by co-expressed Cherry fluorescence.

### L. tarentolae cultivation conditions and induction of protein expression

The fE1E2 and tE1E2 proteins were expressed using the inducible integrative LEXSY expression system according to the manufacturer’s instructions (user’s guide EGE-1400, Jena Bioscience, Jena, Germany). Briefly, the plasmids encoding E1E2 genes were transfected into *L. tarentolae* cells by electroporation. The transfected cells were selected with bleomycin in the suspension culture. The recombinant cell lines were cultivated in 25 cm^2^ tissue culture flasks filled with 10 mL of selective BHI medium supplemented with hemin and antibiotics, at 26 °C. For the protein induction process, the cell culture of recombinant parasites was scaled up using 1L Erlenmeyer flasks filled with 400 ml of BHI medium containing tetracycline (15 μg/ml). The cells were grown for 72 h at 26 °C in agitated culture to a final optical density, at 600 nm (OD_600_) of 4–5.

### Immunofluorescence

For the immunofluorescence labeling, tetracycline-induced *L. tarentolae* cells were washed with PBS and fixed in 4% paraformaldehyde for 30 min at RT. Lysine-coated glass coverslips were covered with fixed cell suspension and left to dry at RT. Next, the cells were permeabilized with 0.5% Triton X-100 in PBS for 10 min at RT. Subsequently, the coverslips were incubated with the primary mouse anti-E1 antibodies diluted 1:1000 in the PBS buffer for 1h at RT. The coverslips were then washed with PBS and incubated with Alexa Fluor 488-labeled goat anti-mouse secondary antibodies (1:1000) for 1 h at RT. After washing, the coverslips were mounted onto microscope slides with the ProLong Gold antifade reagent.

### SDS-PAGE and western blotting

Analysis of the protein expression and purification was carried out using SDS-PAGE. Samples were run in reducing conditions on 4–12% gradient or 10% Bis-Tris gels using the MOPS SDS running buffer. Coomassie staining was performed using the Coomassie protein assay reagent. For western blotting, the proteins were transferred after electrophoresis onto the PVDF membrane using wet transfer in buffer containing 25 mM Tris-Base, 150 mM glycine, and 10% methanol, pH 8.3. Subsequently, the membranes were blocked overnight at 4 °C with 3% nonfat milk in TBST (TBS buffer, 0.1% (v/v) Tween-20). Following the blocking, the membranes were incubated for 1 h at RT with primary anti-E1 (1:2000) or anti-E2 (AP33 1:2000, ALP98 1:1000) diluted in TBST, washed with TBST, and then incubated with goat anti-mouse secondary AP or HRP- conjugated antibodies (Santa Cruz Biotechnology). The results were developed with the BCIP/NBT or chemiluminescent substrate.

### Analysis of N-glycosylation

The protein samples were divided into two equal groups: one for digestion with PNGase F, and one for undigested control. The digestions were carried out O/N in native conditions at 37 °C, in the buffer provided by the manufacturer (New England Biolabs). The digested samples and the controls were analyzed by western blotting using anti-E2 antibodies as described above.

### CD-81 pull-down assay

A recombinant large extracellular loop of human CD81 (CD81-LEL) fused to glutathione S-transferase (GST) was preadsorbed onto glutathione–agarose beads (Sigma). After equilibration with the GST-A buffer (50 mM Tris-HCl pH 8.0, 1 mM EDTA, 80 mM NaCl), 50 μl of the *L. tarentolae* cells lysates containing recombinant E1E2 proteins were added to 30 μl of resin and incubated for 16 h at 4 °C. After incubation, the beads were washed four times with the GST-A buffer and once with water before resuspension in 50 μl of the SDS-PAGE sample buffer with the reducing agent DTT. 30 μl of the sample eluted from glutathione–agarose resin was separated by SDS-PAGE followed by western blotting with anti-E2 (AP33+ALP98), anti-E1 (Santa Cruz) and anti-CD81 (Santa Cruz) primary antibodies diluted 1:1000. The beads not coated with CD81 were used as a negative control and any unspecific signal was not detected. As a positive control E1E2 complex (isolate H77) expressed in HEK cells was used. The CD81-LEL-GST plasmid was constructed in our laboratory by M. Rychłowska.

### Protein purification

#### Purification of the fE1E2 from the cell lysate

The cells from 400 ml of the culture were pelleted at 4 °C, 5000 rpm for 15 min and lysed with 20 ml of ice-cold PBS buffer containing 1% Triton X-100 and a protease inhibitor cocktail (Roche). The fE1E2 with Twin-Strep tag was purified by affinity chromatography from the lysate supernatant using a StrepTactin column according to the manufacturer’s instruction (IBA, Goettingen, Germany). Briefly, the column-bound fE1E2 was eluted in six 0.5 ml fractions with the buffer containing 2.5 mM desthiobiotin. The elution fractions were analyzed by SDS-PAGE Coomassie staining using R-250, western blotting with anti-E2 antibodies, and the Bradford assay.

#### Purification of tE1E2 from the culture medium

The histidine-tagged recombinant tE1E2 complex was purified by affinity chromatography from the culture medium using a Nickel-agarose column (Qiagen). Briefly, the cell culture medium containing the tE1E2 protein was dialyzed against the PBS pH-8.0 buffer and incubated O/N with Nickel-agarose resin in the presence of 10 mM imidazole. Subsequently, the material was loaded onto the column and washed in PBS with 20 mM imidazole. The elution of the recombinant complex was performed at pH-8.0 in the PBS buffer containing 300 mM imidazole. The elution fractions were analyzed by SDS-PAGE Coomassie staining using R-250, western blotting using anti-E2 antibodies, and the Bradford assay. Finally, the fractions with purified protein were pooled and dialyzed against the PBS buffer using Amicon Ultra centrifugal units cut-off 30 K (Merck).

### GNA ELISA

To study whether the recombinant proteins would bind to *Galanthus nivalis* lectin (Sigma), cell lysates containing fE1E2 and tE1E2 in 5-fold dilutions were placed on the ELISA wells precoated with lectin (5 μg/ml). The binding to the lectin was determined using anti-E2 AP33 (1:2000) or ALP98 (1:1000) antibody followed by incubation with goat anti-mouse HRP-conjugated secondary antibodies diluted 1:2000 (The Jackson Laboratory) and the tetramethylbenzidine (TMB) substrate. The reaction was stopped with 0.5 M H_2_SO_4_ and the signal intensity was measured at 450 nm.

To study the conformational epitopes, E1E2 complexes were captured onto the ELISA wells precoated with *G. nivalis* lectin. The binding of the conformational antibodies against HCV: CBH-7, CBH-4G, CBH-4B, HC-1, HC-11, and R04 (kindly provided by Steven Foung, Stanford, USA) and H48, H53 (kindly provided by Jean Dubuisson, Lille, France) was detected by anti-species HRP-conjugated secondary antibodies diluted 1:2000 (Santa Cruz) and the TMB substrate. Huh 7.5 cells electroporated with gt1a/2a chimeric RNA of HCV (H77/C3 plasmid kindly provided by Ralf Bartenschlager, Heidelberg, Germany) were used as the control.

To evaluate the antibody titers of the mouse sera collected 14 days after the last immunization, the fE1E2 and tE1E2 antigens were captured onto the ELISA wells precoated with *G. nivalis* lectin. The 96-well plates were blocked in PBST with 5% BSA. The mouse sera were tested in 5-fold dilutions from 1:1000 to 1:3125000 in PBS containing 0.3% BSA. The binding of the antibodies to the recombinant proteins was detected by goat anti-mouse HRP-conjugated secondary antibodies diluted 1:2000 (Santa Cruz) and the TMB substrate.

For the testing of the sera cross-reactivity, HEK 293 cells were transfected with plasmids expressing E1E2 glycoproteins, derived from different HCV genotypes: gt 1a (H77.20); gt 1b (UKN1B12.6); gt 2a (UKN2A.2.4); gt 3a (UKN31.9); gt 4 (UKN4.21.16); gt 5 (UKN5.14.4); and gt 6 (UKN6.5.8). The plasmids coding for different HCV isolates were kindly provided by Jonathan Ball (Nottingham, UK). The GNA ELISA was performed as described above.

### Immunization of mice

Two groups of six female BALB/c mice, 6–8 weeks of age were immunized subcutaneously with the antigen-adjuvant mixture on days 0, 21, and 42. The antigen for immunization was quantified by the Quick Start Bradford Protein Assay (Bio-Rad). 10 μg of protein for the primary vaccination and 5 μg for the booster vaccinations was mixed immediately before injection with a squalene-based oil-in-water nanoemulsion adjuvant (Addavax, InvivoGen). Mice used as negative controls were immunized with a PBS-adjuvant mixture. Two weeks after the last immunization, the mice were sacrificed and the serum collected for antibody response analysis. All experiments on animals were conducted by an accredited company (Tri-City Academic Laboratory Animal Centre, Medical University of Gdańsk), in accordance with the valid guidelines for animal experimentation. The protocols were approved by the Committee on the Ethics of Animal Experiments of the Medical University of Gdańsk (Permit Number: 46/2012). All surgery was performed under isoflurane anesthesia, and all efforts were made to minimize suffering.

### Virus production and neutralization assay

HCVcc were generated as previously described^48^,^49^. Briefly, RNA was transcribed *in vitro* from linearized plasmids carrying the full length genomic cDNA sequence of the chimeric HCV genotype 2a (plasmid pFK-Jc1 kindly provided by Ralf Bartenschlager, Germany), using T7 RNA Polymerase (Ambion) and electroporated into Huh-7.5 cells. Seventy-two hours post-electroporation the virus-containing supernatant was filtered through 0.45 μm pore membrane, stored for a few days at 4 °C and used as virus stock in infection assays. Virus titer was determined by infecting Huh-7.5 cells with a series of 5-fold virus dilutions, followed by fixation in methanol and probing with the anti-HCV core protein (Santa Cruz) and a HRP-conjugated anti-mouse IgG antibody. For the HCVcc neutralization assay, Huh 7.5 were seeded at 1.2 × 10^4^ cells/well in a 96-well plate. The following day, virus at MOI of 0.1 was pre-incubated for 2 h with mouse sera in 50 μL of DMEM. Then, the mixture was added to the Huh 7.5 cells and incubated for 3 h. Subsequently, the virus-containing medium was removed and replaced with fresh medium. Infection was allowed to proceed for 72 h before IHC analysis was performed. The number of core-positive cell clusters was counted from at least 6 independent microscope fields^50^.

## Additional Information

**How to cite this article**: Grzyb, K. *et al.* Immunogenicity and functional characterization of *Leishmania*-derived hepatitis C virus envelope glycoprotein complex. *Sci. Rep.* **6**, 30627; doi: 10.1038/srep30627 (2016).
